# Intestinal microbiota as biomarkers for different colorectal lesions based on colorectal cancer screening participants in community

**DOI:** 10.3389/fmicb.2025.1529858

**Published:** 2025-02-07

**Authors:** Gairui Li, Dan Zhao, Binfa Ouyang, Yinggang Chen, Yashuang Zhao

**Affiliations:** ^1^Department of Epidemiology, School of Public Health, Harbin Medical University, Harbin, China; ^2^Shenzhen Nanshan Center for Chronic Disease Control, Shenzhen, Guangdong, China; ^3^National Cancer Center/National Clinical Research Center for Cancer/Cancer Hospital & Shenzhen Hospital, Chinese Academy of Medical Sciences and Peking Union Medical College, Shenzhen, China

**Keywords:** intestinal microbiota, colorectal cancer screening, adenoma, colorectal lesions, 16S rRNA

## Abstract

**Introduction:**

The dysregulation of intestinal microbiota has been implicated in the pathogenesis of colorectal cancer (CRC). However, the utilization of intestinal microbiota for identify the lesions in different procedures in CRC screening populations remains limited.

**Methods:**

A total of 529 high-risk individuals who underwent CRC screening were included, comprising 13 advanced adenomas (Aade), 5 CRC, 59 non-advanced adenomas (Nade), 129 colon polyps (Pol), 99 cases of colorectal inflammatory disease (Inf), and 224 normal controls (Nor). 16S rRNA gene sequencing was used to profile the intestinal microbiota communities. The Gut Microbiota Health Index (GMHI) and average variation degree (AVD) were employed to assess the health status of the different groups.

**Results:**

Our findings revealed that the Nor group exhibited significantly higher GMHIs and the lowest AVD compared to the four Lesion groups. The model incorporating 13 bacterial genera demonstrated optimal efficacy in distinguishing CRC and Aade from Nor, with an area under the curve (AUC) of 0.81 and a 95% confidence interval (CI) of 0.72 to 0.89. Specifically, the 55 bacterial genera combination model exhibited superior performance in differentiating CRC from Nor (AUC 0.98; 95% CI, 0.96-1), the 25 bacterial genera combination showed superior performance in distinguishing Aade from Nor (AUC 0.95). Additionally, the 27 bacterial genera combination demonstrated superior efficacy in differentiating Nade from Nor (AUC 0.82). The 13 bacterial genera combination exhibited optimal performance in distinguishing Inf from Nor (AUC 0.71).

**Discussion:**

Our study has identified specific microbial biomarkers that can differentiate between colorectal lesions and healthy individuals. The intestinal microbiota markers identified may serve as valuable tools in community-based CRC screening programs.

## Introduction

Colorectal cancer (CRC) is the third most common cancer and the second leading cause of cancer-associated death worldwide (Eng et al., 2024; Morgan et al., 2023). The incidence of CRC has been on the decline in affluent regions, largely attributed to the implementation of robust screening initiatives. However, this trend has not been uniform across all regions, many low- and middle-income countries, including China, are experiencing an upward trend of CRC incidence (Krul et al., 2023; Wong et al., 2021; Arnold et al., 2017). In addition, there is a notably increase in the mortality and burden of CRC in China, as evidenced by studies indicating a significant rise in cases among individuals in urban regions (Cao et al., 2021; Yu et al., 2018). This epidemiological trend underscores the critical need for targeted screening and early detection strategies to reduce the burden of CRC.

Among CRC screening populations, inflammation, polyps, adenomas, and CRC represent the most prevalent types of colorectal lesions (Gupta, 2022; Chen et al., 2019). A comprehensive meta-analysis has revealed that, among individuals at average risk, the prevalence rates for polyps, non-advanced adenomas, advanced adenomas, and CRC are 30.2, 17.7, 5.7, and 0.3%, respectively (Heitman et al., 2009). Several studies indicate that approximately 50–70% of CRC cases are linked to the progression of adenomas (Morgan et al., 2023; Elsayed et al., 2021; Zauber et al., 2012), and the transformation of an adenoma into CRC typically spans several years to decades (Keum and Giovannucci, 2019; Stryker et al., 1987). Therefore, timely intervention for inflammatory conditions and early removal of polyps and adenomas can prevent disease progression and reduce the incidence and mortality of CRC (He et al., 2020).

The composition of the microbiota has been extensively investigated for its role in various diseases (Sun et al., 2024; Gomaa, 2020). Notably, the gut microbiota has received considerable attention in the context of CRC diagnosis due to its complex involvement in carcinogenesis and tumor progression (Coker et al., 2022; Rezasoltani et al., 2018). Researchers have found that CRC patients have a reduced diversity and richness of gut microbiota compared to healthy individuals (Li et al., 2022; Mo et al., 2020; Cheng et al., 2020). Moreover, fecal microbiota profiles have been explored as potential screening tools for the early diagnosis of CRC, with particular emphasis on candidate pathogens such as *Fusobacterium*, *Parvimonas*, *Gemella, Leptotrichia,* and numerous other microbial taxa. However, the effectiveness of these biomarkers vary significantly depending on the different stage of colorectal lesions, as indicated by studies that highlight the differences in microbial profiles between healthy individuals, adenoma patients, and those with cancer (Tito et al., 2024; Li et al., 2022; Mizutani et al., 2020). Moreover, while some studies have reported promising results in differentiating colorectal adenoma from CRC using microbial biomarkers, further validation is necessary to ensure their reliability and specificity in clinical settings (Guo et al., 2024). Due to the complexity of intestinal microbes, stable early-stage biomarkers are lacking, studies show inconsistent results, and much work remains for their clinical use in CRC screening (Wang et al., 2023; Hua et al., 2022; Olovo et al., 2021; Hale et al., 2017; Zackular et al., 2013). Therefore, their accuracy needs validation across different precancerous stages, and their efficiency should be assessed.

In this community-based real-world study, we aim to understand the microbiota signatures in CRC carcinogenesis. Specifically, we systematically evaluated the microbiota characteristics in individuals at various lesion stages participating in CRC screening programs using 16S rRNA sequencing. By doing so, we aim to not only gain insight into the role of microbiota in colorectal carcinogenesis, but also to identify potential microbiota biomarkers for screening lesions for the development of CRC at different stages. The findings of this study will serve as a valuable resource for guiding future non-invasive screening techniques and preventive measures for CRC.

## Materials and methods

### Study population

This study population consisted of community-based individuals enrolled in the CRC screening program administered by the Chronic Disease Prevention and Control Institute of Nanshan District. All participants were residents of Nanshan District, Shenzhen City, Guangdong Province, China, age 45–74 years old. Each participant provided informed consent prior to participating in the project. Trained medical personnel or investigators utilized a pre-established questionnaire assessment system to conduct risk evaluations (National Cancer Center, China, 2021; Chinese Medical Association, 2015). During the assessment, demographic data such as age, gender, height, weight, presence of chronic diseases, alcohol drinking history were gathered from the participants (more details are provided in the Supplementary material). Subsequently, a fecal immunochemical test (FIT) test was administered, and the stool samples were collected and stored. We excluded individuals with a prior history of CRC, younger than 45 or older than 75, pregnant women, and those who had taken antibiotics or probiotics within 2 months prior to stool collection. CRC risk was determined by testing positive based on the questionnaire assessment system or FIT, and participants were advised to schedule a colonoscopy within 90 days of the risk assessment.

**Figure 1:**
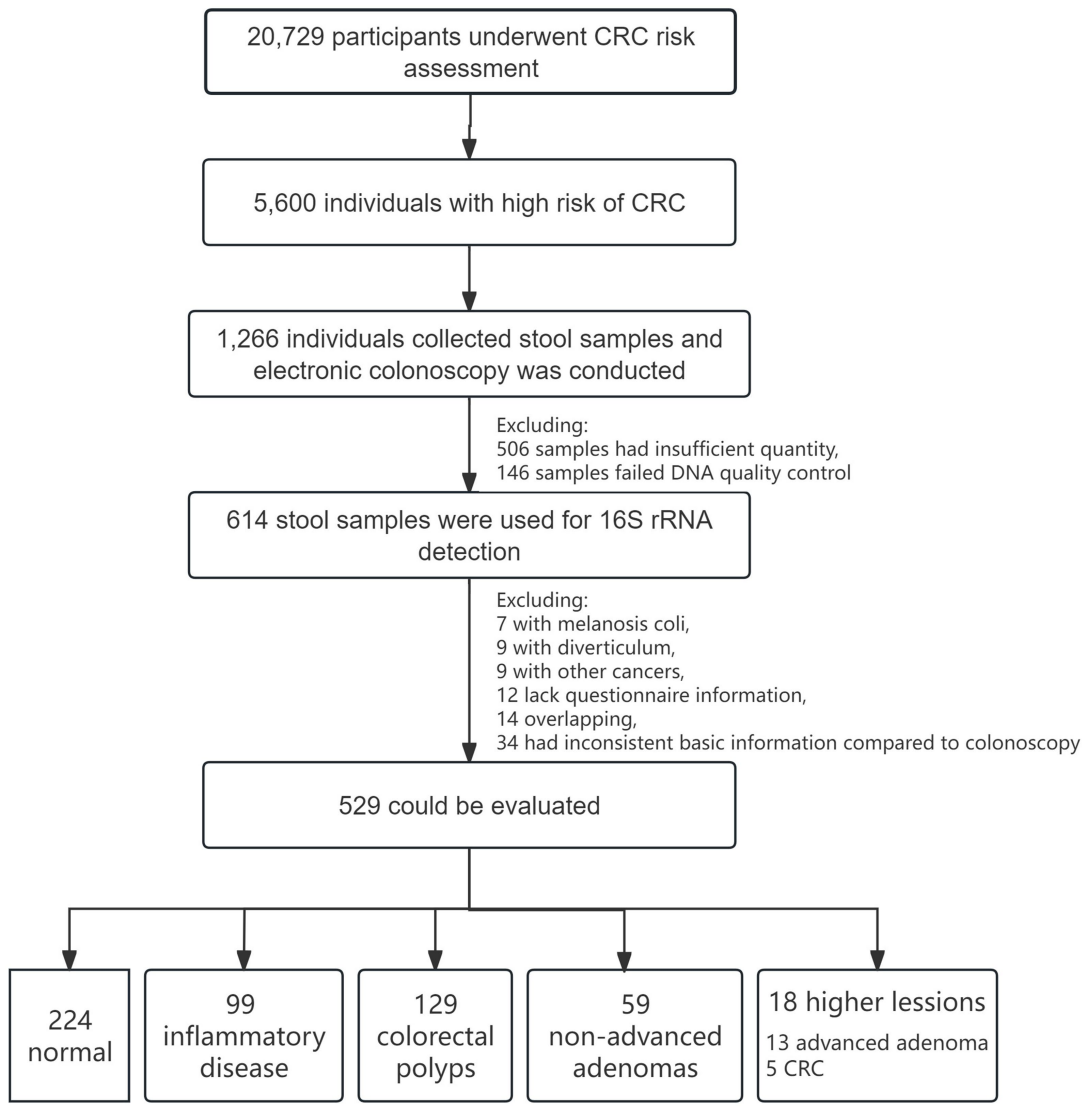
Flowchart of CRC screening cohort and enrollment.

A cohort of 20,729 participants was recruited for CRC screening program between May 2017 and December 2019. Following risk assessment, 5,600 individuals were classified as high risk for CRC. Of these, 1,266 participants provided stool samples before electronic colonoscopy. After excluding 506 samples due to insufficient quantity and 146 samples failing DNA quality control, 614 stool samples were ultimately utilized for 16S rRNA testing. After excluding 7 with melanosis coli, 9 with diverticulum, 9 had other malignant neoplasms (diagnosed within 5 years after the colonoscopy diagnosis), 12 lack questionnaire information, 14 were overlapping and 34 had inconsistent basic information compared to colonoscopy information, a total of 529 samples were analyzed in this study. Among these, 224 subjects were classified as normal, 99 had inflammatory disease, 129 had colorectal polyps, 59 had non-advanced adenomas, and 18 had higher colorectal lesions (13 advanced adenoma, 5 CRC). The flowchart of the cohort enrollment are presented in Figure 1. All participants provided informed consent, and the project was approved by the Ethics Committee of the Shenzhen Nanshan Center for Chronic Disease Control.

### Stool sample collection and storage

Stool sample collection involved two distinct ways. The first involved participants depositing stool sample into collection tubes, which were then stored in a preservation bag with ice packs. These samples were expeditiously transported to a collection site within two-hour, where they were collected by a designated collector, maintained at 2–8°C, and subsequently deposited into a −80°C freezer within four-hour. The alternative way entailed the collection of stool specimens directly into tubes pre-filled with preservation medium. Samples were collected at room temperature by the collector from the designated site where participants had placed them. These were then stored in a −80°C freezer within 24-h. For more detailed information, please refer to the Supplementary Figure 1.

### Colonoscopy and clinical diagnosis

The electronic colonoscopy examination was performed by three tertiary public hospitals in Nanshan District. The normal control (Nor) group was characterized by the absence of inflammation, polyps, or any other bowel diseases observed during colonoscopy. Polyps were confirmed through histopathological if diameter exceeded 0.2 mm. The polyp group (Pol) was these identified as hyperplastic polyp or polyps with diameter less than 0.2 mm. Non-advanced adenoma (Nade) was defined as tubular adenoma with diameter less than 1 cm and lacking villous tissue. Advanced adenoma (Aade) was characterized by villous or tubulovillous features, or serrated adenomas, or adenomas larger than 1 cm, or the identification of high-grade dysplasia via histopathological examination. CRC patients were diagnosed through colonoscopy and histopathological examination. In certain analyses conducted within this study, we categorized the CRC and Aade subgroups as higher lesions (HLes). Based on the location of the polyps, participants were stratified into proximal and distal cases. Proximal polyps were defined as those in the cecum, ascending colon, hepatic flexure, and transverse colon. In contrast, distal polyps are found in the descending colon, the sigmoid colon, the rectum, or in the spleen. Those who exhibited proximal as well as distal polyps being categorized as proximal + distal.

### DNA extraction, library construction and sequencing

Genomic DNA was extracted from stool samples utilizing the Apostle MiniGenomics Genomic DNA Extraction according to the manufacturer’s instructions. The quality and concentration of the DNA were evaluated through the use of 2% agarose gel (Tanon, China) and NanoDrop spectrophotometer (Thermo Fisher Scientific, USA). 16S rRNA gene sequencing was conducted using PCR libraries derived from the bacterial V4 region. The 16S V4 amplicon library using the Acegen 16S V4 Amplicon-Seq Kit. Genomic DNA (1–50 ng) was amplified in the first PCR round (10 cycles) with 16S V4 primers, followed by purification with Agencourt AMPure XP magnetic beads. The purified products underwent a second PCR (15–25 cycles) with index tag primers. The final library was purified again, quantified using Qubit 3.0 and Agilent 2100 Bioanalyzer, and sequenced on the Illumina platform (MiSeq sequencer) with a double-end index sequencing strategy and a read length of PE250. The primers of V4 were as follows:

16S V4 Forward: GACGCTCTTCCGATCTTATGGTAATTG TGTGCCAGCMGCCGCGGTAA.

16S V4 Reverse: TGTGCTCTTCCGATCTAGTCAGTCAGCCGGACTACH VGGGTWTCTAAT.

PCR Forward: AATGATACGGCGACCACCGAGATCTACACTCTTTCC CTACACGACGCT CTTCCGATCT.

PCR Reverse: CAAGCAGAAGACGGCATACGAGATXXXXXXXXXXXXGTGACTGGAGTTCAGACGTGTGCTCTTCCGATCT.

### Sequencing data filtering

In this research, the trimming technique was employed to eliminate low-quality sequencing data and sequencing adapters, yielding refined data for further analysis. Trimmomatic software was utilized for the initial data trimming process, with the procedures of data processing involving the removal of low-quality sequences through a sliding window method using a window size of 4 bases. If the mean base quality falls below 15, the sequence is truncated at that particular position. Eliminate sequences with quality scores below 3 at the start and end, as well as those containing undetermined bases. Additionally, remove sequences contaminated with adapters through two methods: trimming the initial portion if the alignment score with the adapter sequence exceeds 7 (approximately 12 bp) and ensuring a base score above 30 in the overlap region between two sequences. Exclude sequences trimmed to less than 36 nucleotides and those unable to form pairs.

### Data clustering and annotation

Data clustering and annotation were performed using the QIIME 2 software package. Double-ended sequencing data was imported into QIIME 2 to form.qza format that QIIME 2 can recognize. Dada2 was used for denoising and merging of paired-end sequences. FeatureTable and FeatureData summaries in QIIME 2 were used to generate representative sequence lists and perform statistical analysis. Sequences were clustered based on 97% similarity to create multiple taxonomic units, with each unit referred to as an Operational Taxonomic Unit (OTU). The representative sequence list generated by QIIME 2 was converted to tsv format, i.e., OTU table, using the biom software package; OTU table was annotated with species using the feature-classifier function in QIIME 2 to obtain taxonomic levels (phylum, class, order, family, genus, species) for each OTU. The dilution curves suggested the sufficiency of the sequencing data volume (Supplementary Figure 2).

### Microbiota health character index and community structure difference

By comparing the relative abundance of two groups of microbial species associated with good and poor health conditions, the Gut Microbiota Health Index (GMHI) was employed to evaluate the propensity for disease; a lower GMHI value corresponds to a diminished health index (Gupta et al., 2020). The bacterial community stability was evaluated by the average variation degree (AVD), which is mainly based on the deviation of the mean OTU relative abundance. The lower AVD values indicates higher microbiome stability (Xun et al., 2021). The two-dimensional Principal Coordinates Analysis (PCoA) scatter plot was used to illustrate the similarities and differences between the normal control and lesion groups. The Bray–Curtis dissimilarity method was utilized to compute the distances between samples, thereby reflecting the degree of aggregation and dispersion within the sample communities. The intergroup differences in Beta diversity are analyzed using within-group intersample distance matrix data, which assesses the variation in community structure between groups. The Kruskal-Wallis test was applied to statistically evaluate differences among multiple groups.

The TwoGroup Welch’s t-test (Zhang et al., 2018; Ye et al., 2017), linear discriminant analysis (LDA) effect size (LEfSe), and Random Forest analysis at the genus level were employed to identify key microorganisms biomarkers. Taxa identified as ‘unclassified’ at the genus level were excluded. These methods were utilized to determine significant differences in abundance between groups and to develop predictive models. Data analysis of the bacterial composition and species differences between groups were conducted using Majorbio Cloud platform (Han et al., 2024).^1^

### Functional prediction analysis

Based on OTUs of the 16S rRNA sequences data, PICRUSt was used to estimate the abundance of functional categories (KEGG/COG analysis). Subsequently, a differential abundance analysis between different groups was conducted by utilizing LEfSe software with LDA score of 2.

### Statistical analysis

Chi-square or Fisher’s Exact tests were employed for qualitative comparisons, while ANOVA was utilized for quantitative analyses, to evaluate demographic characteristics across different groups. For evaluating accuracy of selected differential related OTUs in predicting various stages of colorectal lesions, receiver operating characteristic curves (ROC) and areas under the curve (AUC) were used. R software (Version 4.2.1)^2^ was used to generate the figures. Statistical significance was determined by a *p-*value of 0.05.

## Results

### Basic characteristics of study subjects

Table 1 displays the demographic information of the 529 participants included in the study, consisting of 196 men and 333 women with age ranging from 45 to 75 years. 48.14% of the participants were under 60 years old. Significant differences among the five groups of participants in terms of gender and age were observed (*p* < 0.05).

**Table 1:** Basic demographic and clinical characteristics of the participants.

Variables		Total (*n* = 529)	HLes (*n* = 18)	Nade (*n* = 59)	Pol (*n* = 129)	Inf (*n* = 99)	Nor (*n* = 224)	Statistic	*p*-value
Gender	Male	196 (37.05)	10 (55.56)	19 (32.20)	67 (51.94)	37 (37.37)	63 (28.13)	*χ*^2^ = 23.15	<0.001^*^
	Female	333 (62.95)	8 (44.44)	40 (67.80)	62 (48.06)	62 (62.63)	161 (71.88)		
Age (years)	<60 y	256 (48.39)	9 (50.00)	25 (42.37)	51 (39.53)	43 (43.43)	128 (57.14)	*χ*^2^ = 12.77	0.012^*^
	> = 60 y	273 (51.61)	9 (50.00)	34 (57.63)	78 (60.47)	56 (56.57)	96 (42.86)		
Marriage	Married	502 (94.90)	17 (94.44)	54 (91.53)	124 (96.12)	96 (96.97)	211 (94.20)	*χ*^2^ = 2.90	0.575
	Separated or single	27 (5.10)	1 (5.56)	5 (8.47)	5 (3.88)	3 (3.03)	13 (5.80)		
Education	<High school	385 (72.78)	12 (66.67)	42 (71.19)	91 (70.54)	66 (66.67)	174 (77.68)	*χ*^2^ = 5.32	0.256
	High school and above	144 (27.22)	6 (33.33)	17 (28.81)	38 (29.46)	33 (33.33)	50 (22.32)		
BMI, Mean ± SD		23.57 ± 3.14	24.45 ± 3.23	23.27 ± 3.62	24.20 ± 3.16	23.21 ± 2.89	23.38 ± 3.06	*F* = 2.32	0.056
Alcohol history	Yes	92 (17.39)	3 (16.67)	10 (16.95)	33 (25.58)	14 (14.14)	32 (14.29)	*χ*^2^ = 8.27	0.082
	No	437 (82.61)	15 (83.33)	49 (83.05)	96 (74.42)	85 (85.86)	192 (85.71)		
Diarrhea	Yes	116 (21.93)	2 (11.11)	9 (15.25)	26 (20.16)	30 (30.30)	49 (21.88)	*χ*^2^ = 7.06	0.133
	No	413 (78.07)	16 (88.89)	50 (84.75)	103 (79.84)	69 (69.70)	175 (78.12)		
Astriction	Yes	158 (29.87)	5 (27.78)	22 (37.29)	40 (31.01)	29 (29.29)	62 (27.68)	*χ*^2^ = 2.20	0.700
	No	371 (70.13)	13 (72.22)	37 (62.71)	89 (68.99)	70 (70.71)	162 (72.32)		
Mucous	Yes	155 (29.30)	7 (38.89)	19 (32.20)	35 (27.13)	31 (31.31)	63 (28.12)	*χ*^2^ = 1.67	0.795
Bloody stool	No	374 (70.70)	11 (61.11)	40 (67.80)	94 (72.87)	68 (68.69)	161 (71.88)		
Appendicitis	Yes	59 (11.15)	4 (22.22)	7 (11.86)	11 (8.53)	9 (9.09)	28 (12.50)	*χ*^2^ = 3.99	0.408
	No	470 (88.85)	14 (77.78)	52 (88.14)	118 (91.47)	90 (90.91)	196 (87.50)		
Cholecystitis	Yes	98 (18.53)	1 (5.56)	13 (22.03)	18 (13.95)	16 (16.16)	50 (22.32)	*χ*^2^ = 6.78	0.148
	No	431 (81.47)	17 (94.44)	46 (77.97)	111 (86.05)	83 (83.84)	174 (77.68)		
Psychosis	Yes	134 (25.33)	5 (27.78)	19 (32.20)	36 (27.91)	24 (24.24)	50 (22.32)	*χ*^2^ = 3.12	0.538
	No	395 (74.67)	13 (72.22)	40 (67.80)	93 (72.09)	75 (75.76)	174 (77.68)		
Hypertension	Unknow	70 (13.23)	2 (11.11)	10 (16.95)	17 (13.18)	15 (15.15)	26 (11.61)	*χ*^2^ = 9.83	0.277
	Yes	121 (22.87)	7 (38.89)	16 (27.12)	35 (27.13)	22 (22.22)	41 (18.30)		
	No	338 (63.89)	9 (50.00)	33 (55.93)	77 (59.69)	62 (62.63)	157 (70.09)		
Cancer history	No	499 (94.33)	18 (100.00)	55 (93.22)	120 (93.02)	97 (97.98)	209 (93.30)	*χ*^2^ = 4.54	0.338
	Yes	30 (5.67)	0 (0.00)	4 (6.78)	9 (6.98)	2 (2.02)	15 (6.70)		
Diabetes	Unknow	70 (13.23)	2 (11.11)	10 (16.95)	17 (13.18)	15 (15.15)	26 (11.61)	*χ*^2^ = 8.01	0.432
	Yes	57 (10.78)	2 (11.11)	9 (15.25)	19 (14.73)	10 (10.10)	17 (7.59)		
	No	402 (75.99)	14 (77.78)	40 (67.80)	93 (72.09)	74 (74.75)	181 (80.80)		
FIT	Negative	419 (79.21)	10 (55.56)	51 (86.44)	100 (77.52)	82 (82.83)	176 (78.57)	*χ*^2^ = 9.05	0.060
	Positive	110 (20.79)	8 (44.44)	8 (13.56)	29 (22.48)	17 (17.17)	48 (21.43)		
Polyp number	1	110 (53.40)	10 (55.56)	33 (55.93)	67 (51.94)	/	/	*χ*^2^ = 0.30	0.862
	>1	96 (46.60)	8 (44.44)	26 (44.07)	62 (48.06)	/	/		
Lesion site	Proximal	54 (26.21)	5 (27.78)	18 (30.51)	31 (24.03)	/	/	–	0.66
	Distal	114 (55.34)	8 (44.44)	31 (52.54)	75 (58.14)				
	proximal + distal	38 (18.45)	5 (27.78)	10 (16.95)	23 (17.83)	/	/		

F, ANOVA; χ^2^, Chi-square test; −, Fisher exact; SD, standard deviation. ^*^p-value < 0.05.

### Taxonomic classification of microbial communities

Based on the initial analysis, 28,952 *de novo* OTUs were categorized into 19 phyla, 33 classes, 95 orders, 175 families, and 503 genera. By including only taxa with at least two sequence reads in at least five participants, 14 phyla, 21 classes, 58 orders, 112 families, 347 genera, and 3,560 OTUs remain. And there were 1,046 OTUs shared among the Les, Nade, Inf, Pol, and Nor groups in addition to group-specific unique ones. (The distribution of OTUs among different groups is illustrated in Supplementary Figure 3).

### Intestinal microbiota health and diversity analysis

**Figure 2:**
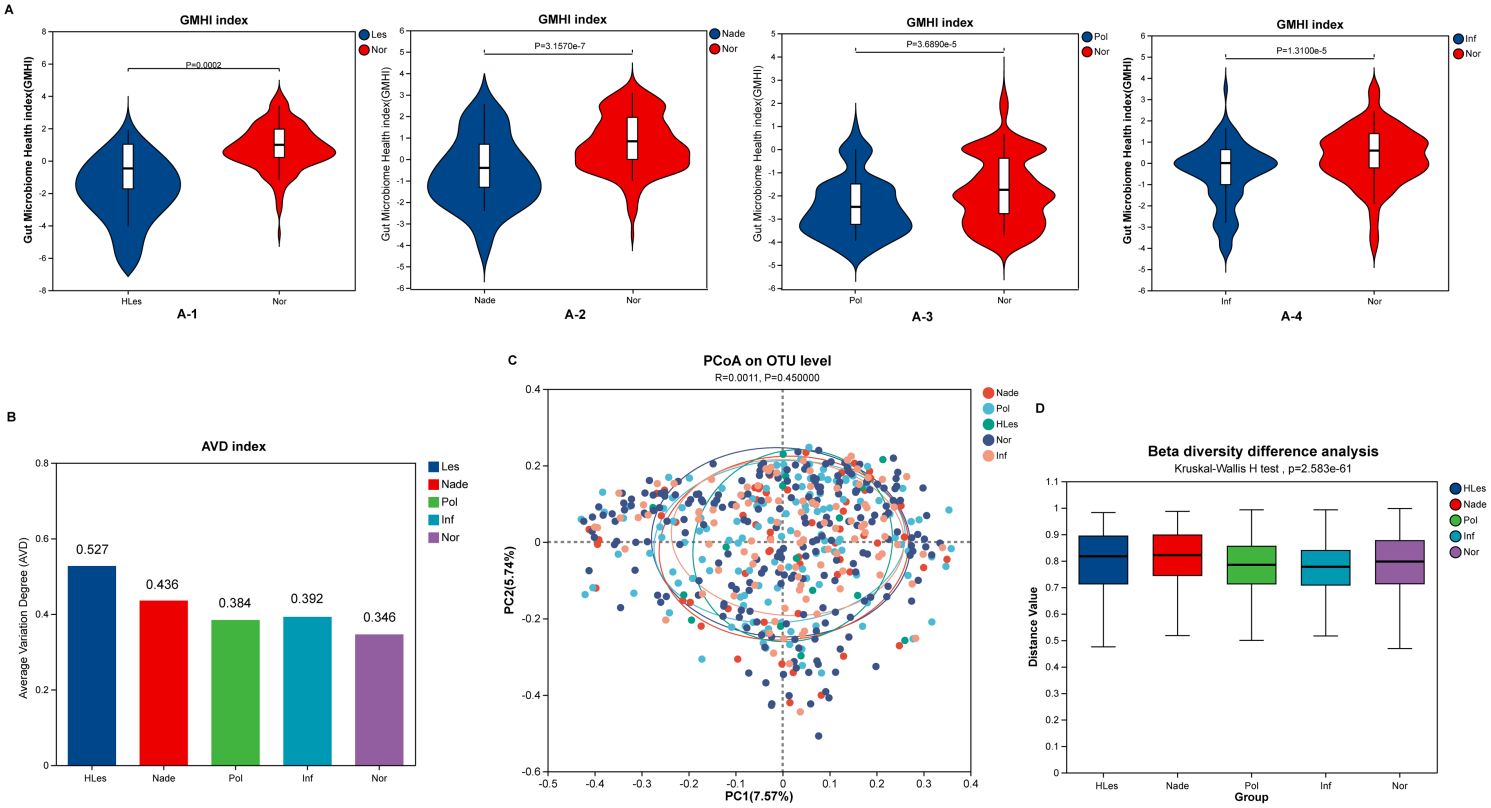
Microbial diversity and health index of the five groups. **(A)** Comparing of GMHI between Nor group with the other four lesion groups. **(A-1)** GMHI of HLes and Nor groups. **(A-2)** GMHI of Nade and Nor groups. **(A-3)** GMHI of Pol and Nor groups. **(A-4)** GMHI of Inf and Nor groups. **(B)** AVD of these five groups. **(C)** PCoA plot of these five groups. **(D)** Intergroup difference in beta diversity.

In contrast to HLes, Nade, Inf, and Pol groups, the Nor control group had significantly higher GMHIs (Wilcoxon rank sum test, *p* < 0.05; Figure 2A). The HLes group had the highest AVD of 0.527, and the control group had the lowest AVD of 0.346 (Figure 2B). The PCoA scatter plot indicates no significant differences in the overall community structure of beta diversity among the five groups (Bray-Curtis; *p* = 0.477) (Figure 2C). Furthermore, the analysis of intergroup differences in beta diversity based on within-group intersample distance matrix data shows significant differences in community structure among the five groups (Bray-Curtis; Kruskal-Wallis H test, *p* = 2.583e-61) (Figure 2D).

### Community composition of microbiota in different groups

**Figure 3:**
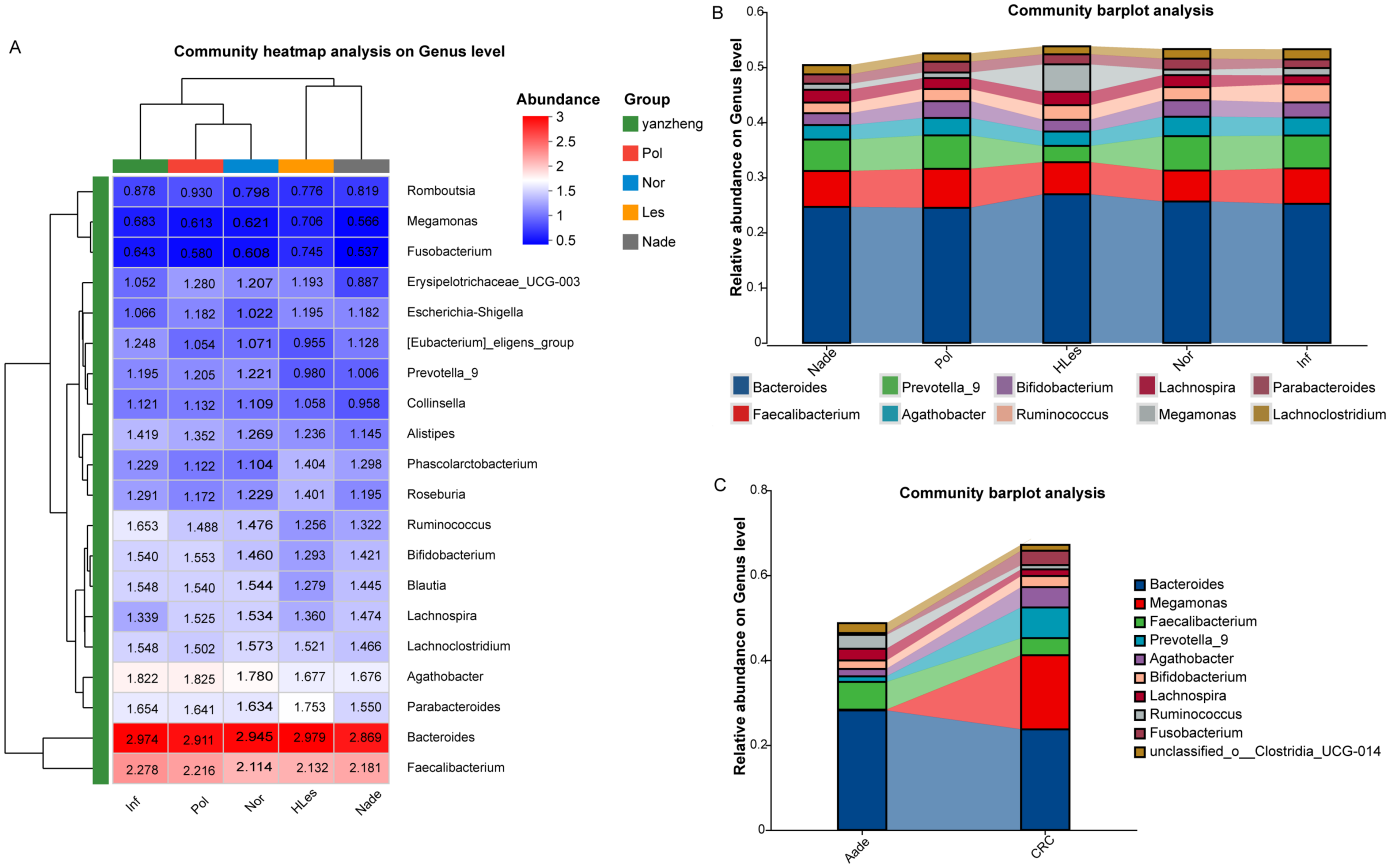
Community heatmap and barplot analysis at genus level within the five groups. **(A)** Community heatmap of these five groups (top 50). **(B,C)** Community barplot of the different groups.

Figure 3A heatmap illustrates the bacterial relative abundances which were log10 transformed of the 30 most dominant bacterial communities at the genus level across the five groups. The predominant genera within the top 10 abundances collectively constitute over 50% of the total sequences in these groups. Among these, *Bacteroides* and *Faecalibacterium* emerge as the two most dominant genera. Additionally, *Megamonas* was identified as the third-ranked (4.98%) prevalent genus in the HLes group (Figure 3B and Supplementary Figure 5). Further analysis revealed that in the CRC group, *Megamonas* constituted 17.44% of the microbial community, ranking second after *Bacteroides* (23.72%) and followed by *Prevotella_9* (7.24%). Conversely, in the Aade group, *Prevotella_9* represented a relatively minor proportion at 1.29%, with the predominant taxa were *Bacteroides* (28.18%), *Faecalibacterium* (6.53%), and *Ruminococcus* (3.26%) (Figure 3C and Supplementary Figure 5).

### Species differences in different groups

**Figure 4:**
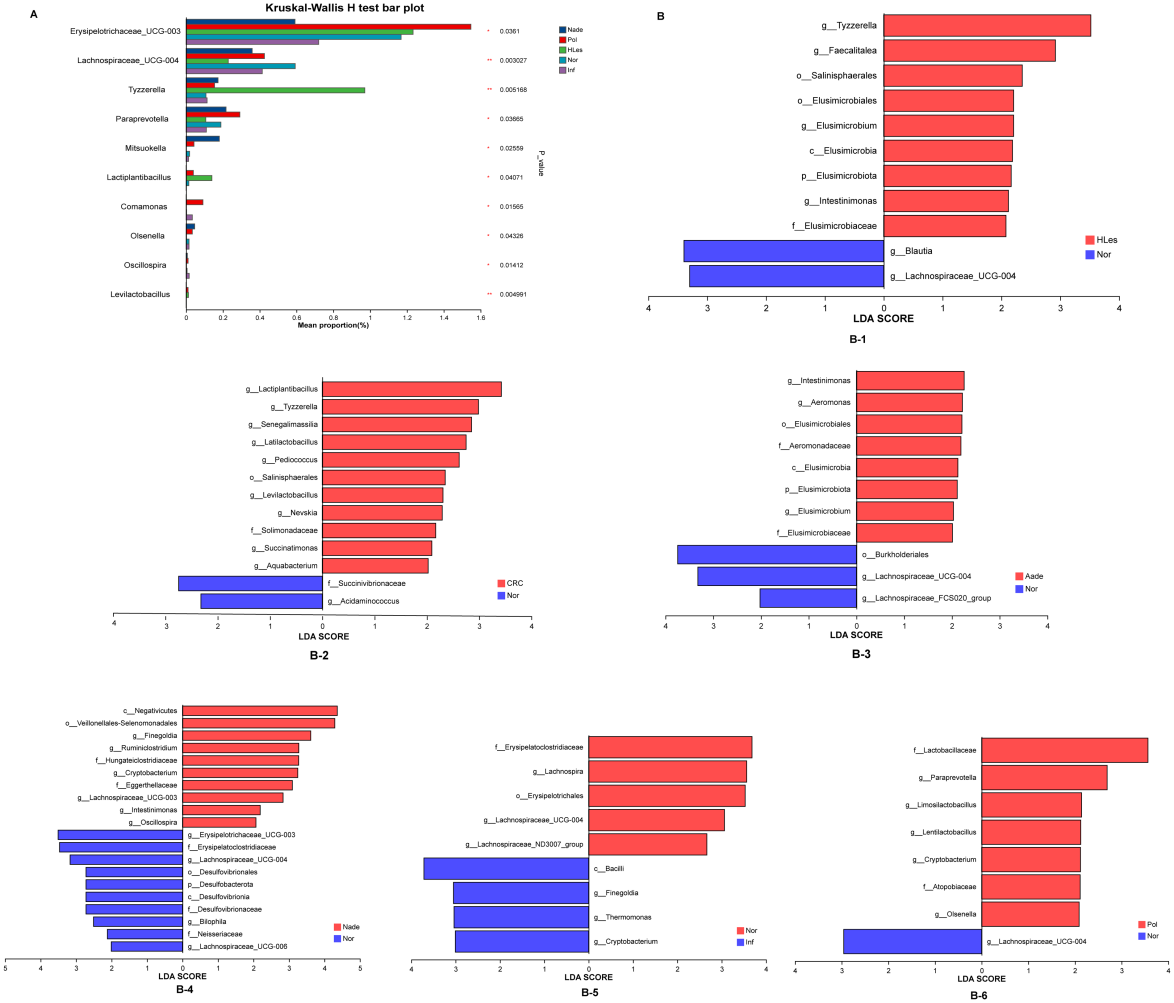
**(A)** Multiple groups significant difference test shows that some species were significantly different at the genus level among the five groups of samples. **(B)** LEfSe analysis shows the differentially abundant bacteria between normal control and lesion groups.

The multiple groups’ significant difference test illustrated in Figure 4A reveals that *Erysipelotrichaceae_UCG-003*, *Lachnospiraceae_UCG-004*, *Tyzzerella*, and other genera, demonstrated statistically significant or highly significant differences in average relative abundance across the five groups (*p* < 0.05, *p* < 0.01, *p* < 0.0001). Differentially abundant fecal bacterial taxa between the disease and control groups were further identified by LEfSe analysis (Figure 4B). The species *Tyzzerella*, *Faecalitalea*, et al. exhibit significant differences between the HLes and Nor groups. More specifically, *Lactiplantibacillus*, *Tyzzerella*, et al. show significant differences between the CRC and Nor groups. And *Intestinimonas*, *Aeromonas*, et al. are significantly different between the Aade and Nor groups. Furthermore, class of *Negatwoutes* and *Vellondlales-Seenomonadales*, et al. exhibit significant differences between the Nade and Nor groups. The species *Ladobaallacese* and *Paraprevotela* exhibit significant differences between the Pol and Nor groups. Aside from that, the species *Eysipdatodotrdlaceae* and *Lschnospira* show significant differences between the Inf and Nor groups.

### Prediction model for distinguish different disease status

**Figure 5:**
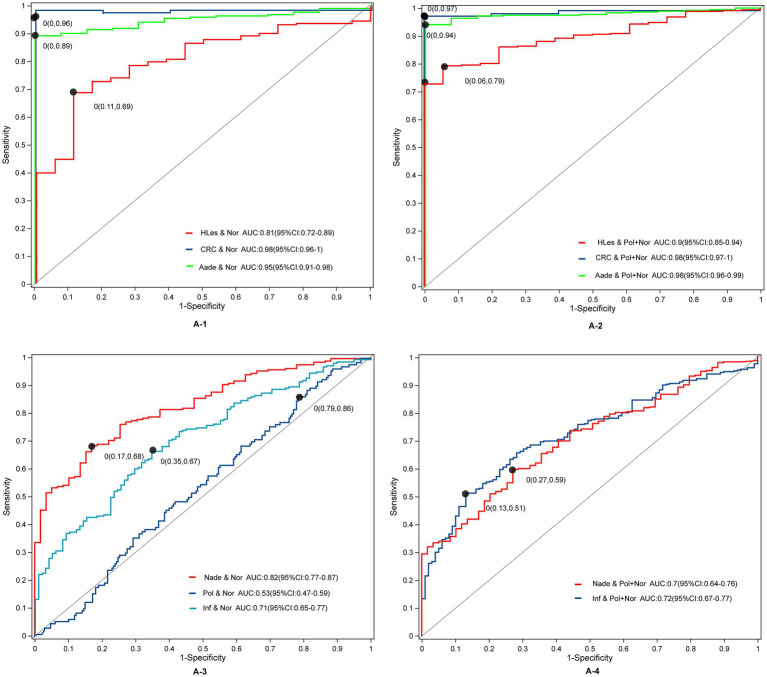
The ROC curves and their corresponding AUCs for microbiota OTU biomarkers among normal and disease groups. **(A-1)** The ROC curves between the HLes and Nor groups. **(A-2)** The ROC curves between the Les and Pol + Nor groups. **(A-3)** The ROC curves between the Nade, Pol, Inf, and Nor groups. **(A-4)** The ROC curves between the Nade, Inf, and Pol + Nor groups.

Through LEfSe, TwoGroups comparison and Random Forest analysis, we construct optimum OTU sets and develop corresponding predictive models to differentiate lesions from healthy controls. After adjusting for the covariates of age and gender and comparing the predictive models developed using these three methodologies, the TwoGroups method exhibit superior efficacy in screening for higher lesions. The AUC values were determined to be 0.81 for the comparison between HLes and Nor, 0.90 for HLes versus Nade, 0.90 for HLes versus Pol, and 0.93 for HLes versus Inf (Figure 5A-1 and Table 2). Following further stratification of the HLes group into the CRC and Aade subgroups, the AUC values for HLes versus Nor increased to 0.98 and 0.95, respectively (see Figure 5A-1). In contrast, when compared to the Nor group, the predictive efficacy of the AUC for the Nade, Inf, and Pol groups was 0.82, 0.71, and 0.53, respectively (refer to Figure 5A-3). Given the low efficiency to distinguish the Pol from the Nor group, individuals from the Pol group were combined with the Nor group to form a control group. Consequently, the AUC values for discriminating the HLes group, the Nade group, and the Inf group from the combined Nor and Pol group were 0.9 (with 0.98 for CRC and 0.98 for Aade), 0.7, and 0.72, respectively, as illustrated in Figures 5A-2, A-4. Table 2 illustrates the predictive performance of the various models in distinguishing between the lesion groups and the corresponding healthy control groups.

**Table 2:** Prediction models’ efficiency using microbiota biomarkers on genus level conferred by Random Forest, LefSe, and TwoGroups methods.

Groups	Methods		
	Random forest	LefSe	TwoGroups
HLes vs. Nor	0.65 (16, 0.48–0.81)	0.54 (15, 0.36–0.71)	0.81 (13, 0.72–0.89)
CRC vs. Nor	/	0.85 (13, 0.68–1.00)	0.98 (55, 0.96–1.00)
Aade vs. Nor	0.61 (7, 0.44–0.78)	0.60 (11, 0.43–0.76)	0.95 (25, 0.91–0.98)
HLes vs. Nade	0.62 (8, 0.46–0.79)	/	0.90 (14, 0.83–0.97)
CRC vs. Nade	/	0.85 (22, 0.57–1.00)	0.98 (41, 0.94–1.00)
Aade vs. Nade	0.69 (13, 0.53–0.84)	0.68 (2, 0.51–0.84)	0.97 (23, 0.93–1.00)
HLes vs. Pol & Nor	0.57 (15, 0.43–0.72)	0.67 (12, 0.51–0.82)	0.90 (24, 0.85–0.94)
CRC vs. Pol & Nor	/	0.81 (7, 0.55–1.00)	0.98 (90, 0.97–1.00)
Aade vs. Pol & Nor	0.54 (30, 0.38–0.70)	0.61 (10, 0.43–0.79)	0.98 (46, 0.96–0.99)
HLes vs. Inf	0.65 (39, 0.51–0.80)	0.76 (26, 0.62–0.90)	0.93 (19, 0.89–0.98)
CRC vs. Inf	/	0.81 (13, 0.51–1)	0.9 (19, 0.84–0.96)
Aade vs. Inf	0.77 (23, 0.64–0.89)	0.7 (15, 0.53–0.87)	0.75 (2, 0.70–0.80)
Nade vs. Inf	0.74 (11, 0.65–0.82)	0.64 (13, 0.55–0.74)	0.61 (6, 0.52–0.7)
Nade vs. Nor	0.61 (21, 0.52–0.7)	0.56 (20, 0.48–0.65)	0.82 (27, 0.77–0.87)
Inf vs. Nor	/	0.59 (9, 0.51–0.66)	0.71 (13, 0.65–0.77)

/, No data; n1 (n2, n3-n4) n1: the AUC value of the prediction model. n2: The top distinctive bacterial genera were identified using corresponding analysis methods based on relative abundances in the control and lesion groups. n3-n4: 95%CI.

### Functional analysis

**Figure 6:**
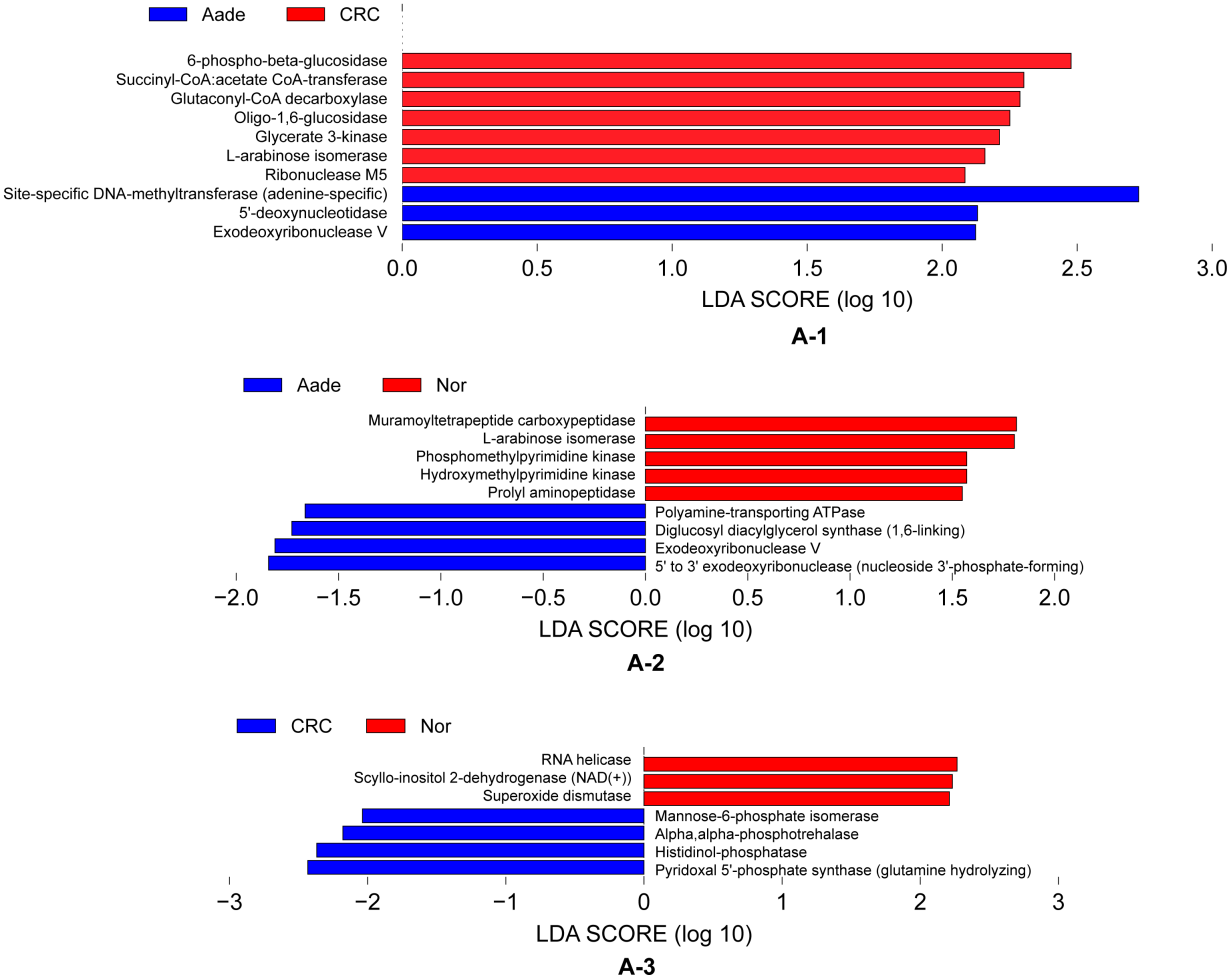
Inferred functional differences between groups of KEGG enzymes using 16S amplicons. **(A-1)** The bars represent enzymes that are predicted to be significantly enriched in the microbiota of individuals with Aade and CRC compared to those in the Inf, Pol, and Nor groups. **(A-2,A-3)** Bule bars represent enzymes predicted to be more prevalent in individuals with Aade and CRC compared to the Nor group. In contrast, red bars denote enzymes that are more commonly found in the Nor group than in those with CRC and Aade.

We detected distinct enzymatic profiles in the groups using PICRUSt2 for functional prediction. Notably, 6-phospho-beta-glucosidase, along with other six enzymes, were more prevalent in individuals with Aade and CRC compared to those in the Inf, Pol, and Nor groups (refer to Figure 6A-1). The microbiota of the Aade group exhibited an elevated presence of polyamine-transporting ATPase, diglucosyl diacylglycerol synthase, exodeoxyribonuclease V, and 5′ to 3′ exodeoxyribonuclease. Conversely, the microbiota of the Nor group was predicted to have an increased biosynthesis of muramoyltetrapeptide carboxypeptidase and other enzymes (see Figure 6A-2 for more information). Additionally, the CRC group’s microbiota demonstrated an increased presence of mannose-6-phosphate isomerase, alpha-phosphotrehalase, histidinol-phosphatase, and pyridoxal 5′-phosphate synthase. In contrast, the microbiota of the Nor group is predicted to show increased biosynthetic activity of RNA helicase and additional enzymes (see Figure 6A-3).

## Discussion

In our study, we observed that the health index of the intestinal microbiota in individuals with colorectal lesions was notably lower compared to that of the healthy control group. We identified potential microbial biomarkers that correlate with different stages of colorectal lesions who were undergoing CRC screening. In addition, a comparison of 16S sequences between disease and control groups revealed possible metabolic changes in microbes.

The examination of intestinal microbiota is increasingly acknowledged as a significant approach for identifying various tumor types (Liu et al., 2024; Amaro-da-Cruz et al., 2024). Previous research has underscored a robust association between gut microbiota and colorectal carcinogenesis, indicating that distinct microbial profiles could serve as potential biomarkers for screening and diagnosis (Tilg et al., 2018; Konstantinov et al., 2013). However, challenges remain in terms of variability in microbiota composition among different individuals and the need for robust methodologies to accurately assess microbial communities in different precancerous stages (Amaro-da-Cruz et al., 2024; Lam and Fong, 2024).

Microbiota dysbiosis has emerged as a significant factor in the development of colorectal carcinogenesis. Research indicates that dysbiosis of the gut microbiota may be implicated in the pathogenesis of CRC, particularly in its early stages (Yu et al., 2023; Kim et al., 2020; Sobhani et al., 2011). In peptic ulcer disease, dysbiosis of the gut microbiota is often observed, which can lead to an imbalance in the release of inflammatory factors such as interleukin-6 (IL-6), interleukin-1β (IL-1β), and tumor necrosis factor-*α* (TNF-α). These factors can impair the mucosal barrier function, promote ulcer formation, and impede healing (Garavaglia et al., 2024). Moreover, the presence of specific bacteria, such as *Helicobacter pylori*, can significantly alter the composition of the gut microbiota, this alteration can further contribute to the development and progression of gastrointestinal cancer (Chen et al., 2021; Dash et al., 2019). Research found that individuals with conventional adenomas exhibited lower species richness in their gut microbiota compared to polyp-free controls, suggesting that specific microbial profiles may be associated with early stages of colorectal carcinogenesis (Peters et al., 2016). Evidence points to a complex interplay between microbiota dysbiosis and colorectal carcinogenesis, where specific microbial changes may contribute to the initiation and progression of cancer (Kang et al., 2023; Pakbin et al., 2023; Coker et al., 2018). Our study revealed a decline in the health index during the early stages of colorectal lesions, with the stability of the microflora being inferior to that observed in the healthy intestinal population participating in community-based CRC screening.

Numerous bacterial species have been found to be associated with the pathogenesis of CRC, such as *Fusobacterium nucleatum*, *Bacteroides fragilis*, *Escherichia coli*, *Enterococcus faecalis*, *Helicobacter hepaticus*, *Peptostreptococcus anaerobius*, *Helicobacter pylori*, *Streptococcus bovis*, and *Porphyromonas gingivalis* (Senthakumaran et al., 2023; Kaźmierczak-Siedlecka et al., 2020; Wong and Yu, 2019; Yu et al., 2017). Our study identified Bacteroides as the predominant genus within our CRC screening cohort. Nonetheless, the variation in the mean relative abundance of this bacterium across different stages of the disease was not pronounced. This finding contrasts with previous research that has recognized *Bacteroides fragilis* as one of the seven bacteria enriched in CRC across diverse populations (Dai et al., 2018). It is possible that the methodological differences between our study and previous research contribute to the observed discrepancy. Research has demonstrated that the composition of the gut microbiota, including Bacteroides, Fusobacterium and others, is correlated with various factors such as transit time, fecal calprotectin levels, and BMI, which are significant covariates in the study (Tito et al., 2024; Baraibar et al., 2023; Hale et al., 2017).

At the genus level, among the top ten average relative abundances of microbial communities, significant alterations in *Megamonas*, *Prevotella_9*, *Tyzzerella, Agathobacter* and others were observed during the process of colorectal carcinogenesis. However, these strains, though relatively uncommon in earlier studies and documented in a limited number of investigations, have been noted in the literature. Notably, *Megamonas* has been reported to be associated with the progression of CRC, particularly showing elevated levels in moderately differentiated CRC cases (Qi et al., 2022; Han et al., 2020). This suggests a potential involvement of *Megamonas* in the progression and severity of CRC. Moreover, *Prevotella*, a bacterium influenced by dietary factors, has been identified as an oral pathogen linked to an increased risk of CRC (Yang et al., 2019). Additionally, research conducted by Zhang et al. (2023) also considers *Prevotella* as a potential biomarker for CRC diagnosis. *Tyzzerella* has been recognized as a predominant bacterial group capable of differentiating between individuals with CRC, adenomas, and those who are healthy. This implies that the presence of *Tyzzerella* in the gut microbiome could potentially serve as an indicator for the development of CRC (Senthakumaran et al., 2023; Kim et al., 2022). Our findings also underscore the important potential of these gut microbiome as biomarkers for assessing colorectal precancerous lesions.

Numerous studies have suggested that biomarkers related to the intestinal microbiota show promise for the early detection of CRC. However, a definitive biomarker for accurately predicting the presence of early colorectal cancerous lesions remains elusive, limiting the use of gut microbiota as a diagnostic tool in CRC screening populations (Olovo et al., 2021; Krigul et al., 2021; Zhou et al., 2020). A variety of populations, along with different sampling and detection methods, may contribute to this problem. This study focused on individuals who were undergoing CRC screening. We utilized three distinct methodologies to identify unique microbiota profiles that showed significant differences between the groups. The microbiota biomarkers identified in our research demonstrated the ability to distinguish advanced adenomas and CRC from healthy controls, with the predictive accuracy of the selected microbial markers achieving the AUC values of 0.98. The AUC values were minimally affected by combining samples from the Pol group with those from the normal group. The predictive performance observed in this study is comparable to the report which employed 11 metabolite biomarkers and six bacterial species to differentiate CRC from normal controls (Coker et al., 2022). Additionally, researchers achieved high AUC values using bacterial-related biomarkers in their analyses to differentiate CRC vs. CRA (AUC 0.994) (Gao et al., 2022) and CRC vs. NC (AUC 0.930) (Yang et al., 2020). Notably, the AUC value for differentiate CRA from NC derived from our study exceed those reported in several other studies, including an AUC value of 0.8 (Wu et al., 2021) and an AUC of 0.792 (Wei et al., 2020). These findings collectively indicate that the gut microbiota biomarkers hold promise for practical application in initiatives for CRC screening and early detection.

Although the bacterial-related biomarkers identified in this study have demonstrated satisfactory results as screening markers within a real-world CRC screening population, several limitations persist. Notably, challenges were encountered in obtaining stool samples from individuals undergoing CRC screening, as not all participants in the screening program provided fecal samples. Consequently, our study was limited to testing the gut microbiota of only those individuals who both underwent colonoscopy and provided fecal samples. Additionally, the current study is limited by a small sample size, particularly within the advanced adenoma and CRC populations. Further research is required to ascertain the applicability of these findings to populations undergoing CRC screening in diverse cultural and regional contexts.

## Conclusion

This study has identified specific microbial biomarkers that can differentiate between colorectal or other site lesions and comparative healthy individuals, thereby advancing our understanding of the potential utility of gut microbiota in community-based CRC screening programs. And efforts will be concentrated on broadening the application of gut microbiota as CRC screening biomarkers across diverse populations.

## Data Availability

The original contributions presented in the study are included in the article/Supplementary material, further inquiries can be directed to the corresponding authors.

## Footnotes

^1^ https://cloud.majorbio.com

^2^ http://www.R-project.org
