# Supplementary material for: Intestinal microbiota as biomarkers for different colorectal lesions based on colorectal cancer screening participants in community
Source: Front Microbiol. 2025 Feb 7;16:1529858. doi: 10.3389/fmicb.2025.1529858 (PMC11844352; doi:10.3389/fmicb.2025.1529858)
Supplement: Supplementary file 1 [file Supplementary_file_1.docx]

**Intestinal Microbiota as Biomarkers for Different Colorectal Lesions Based on Colorectal Cancer Screening Participants in Community**

Gairui Li et al.

**Supplementary Methods**

**Detailed demographic information of the participation**

alcohol drinking history: a history of drinking alcohol on a weekly basis, either in the past or currently, for a minimum duration of one year

Chronic Diarrhea History: Refers to a cumulative duration of diarrhea exceeding three months within the last two years, with each episode lasting more than one week.

Chronic Constipation History: Indicates constipation persisting for more than two months per year over the past two years.

Mucous and/or Bloody Stool History: Describes the presence of visible non-transparent, slightly viscous mucus, or purulent yellow-white opaque mucus on the surface of the stool.

Chronic Appendicitis or Appendectomy History: Pertains to a history of chronic appendicitis or surgical removal of the appendix.

Chronic Cholecystitis or Cholecystectomy History: Pertains to a history of chronic cholecystitis or surgical removal of the gallbladder.

Psychological Trauma or Distress History: Refers to the experience of significant psychological trauma or distress within the past 20 years, such as the death of a spouse, the death of a first-degree relative, personal unemployment, divorce, or other similar events.

**Stool sample collection**


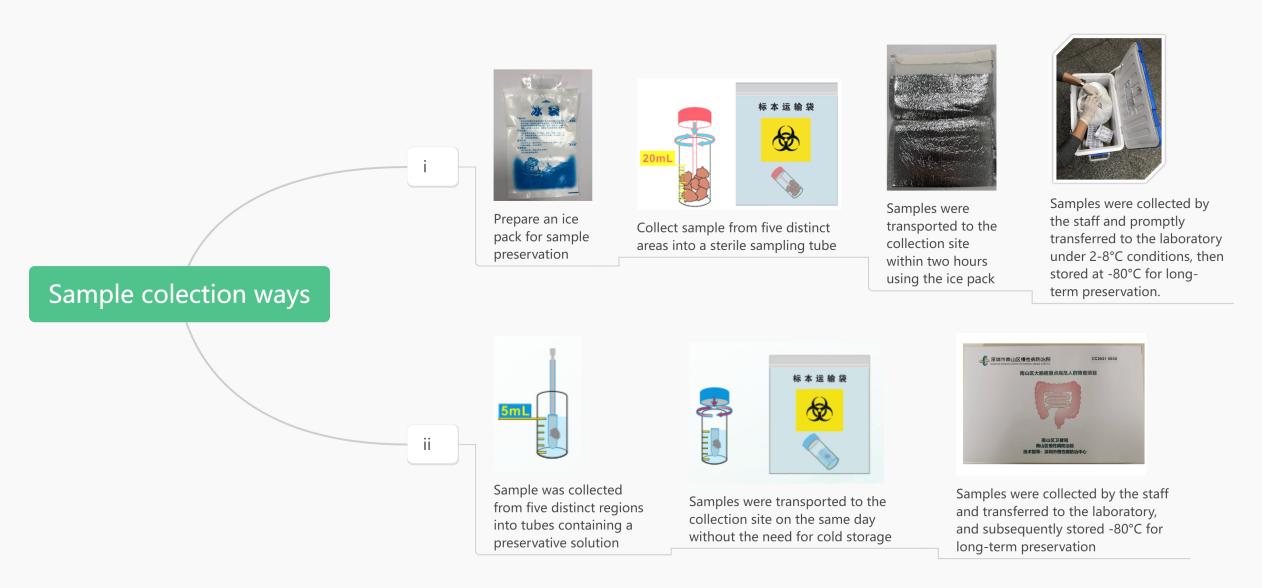


Figure 1 Stool sample collection ways

**Supplementary Results**


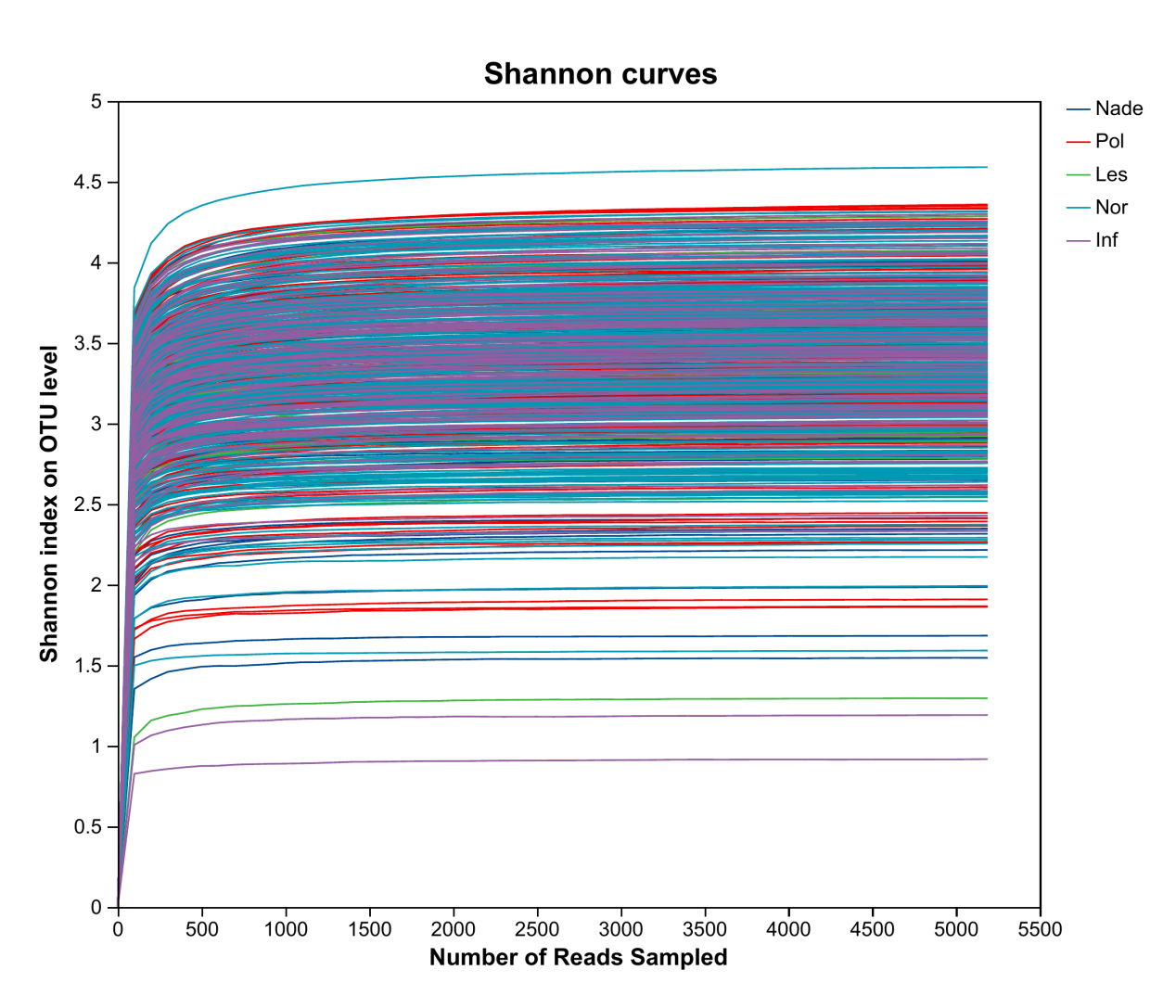


Figure 2 Shannon curves of all participants based on OUT level


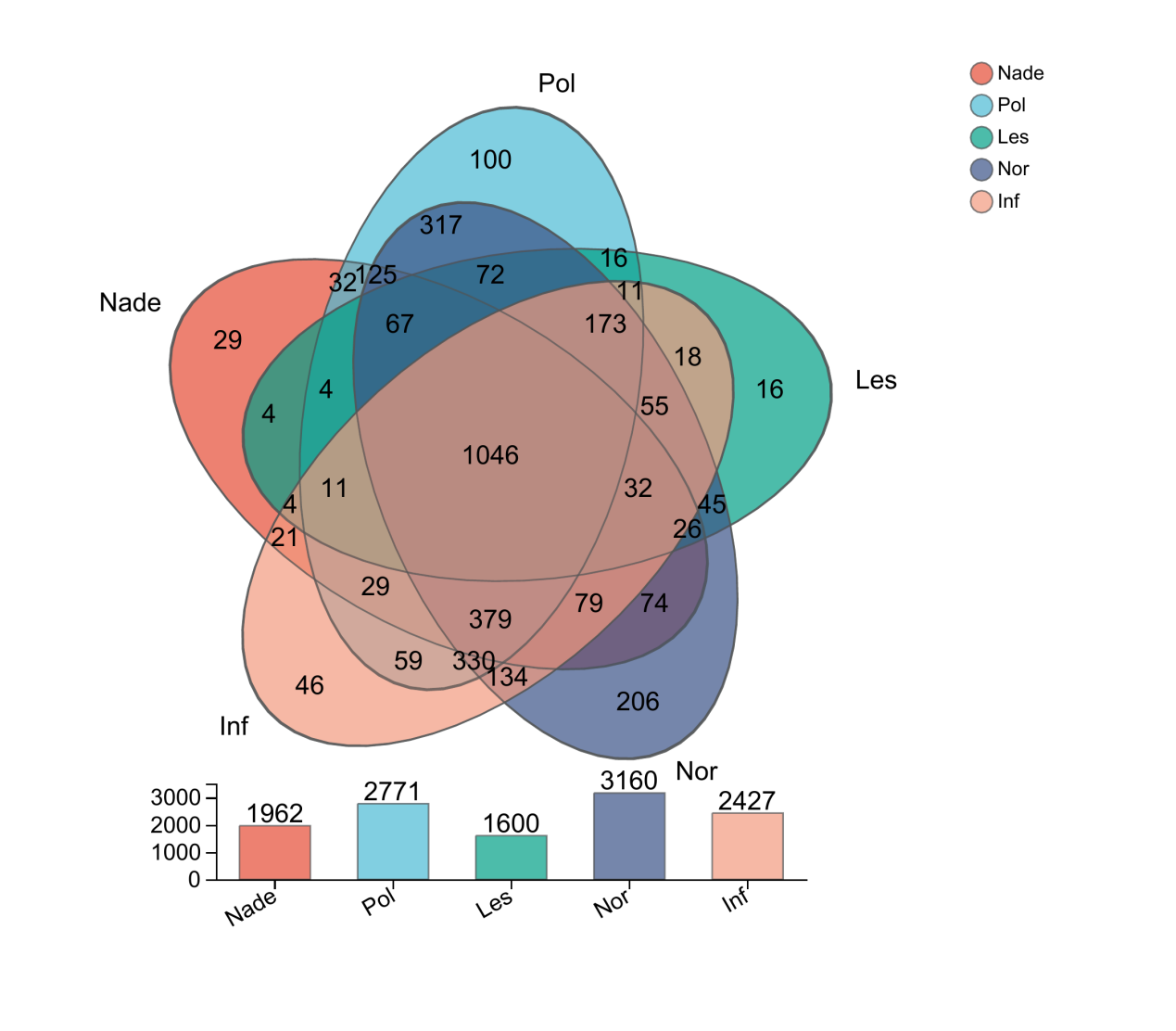
Figure 3 Venn diagram: distribution of intestinal bacteria OTUs among different groups


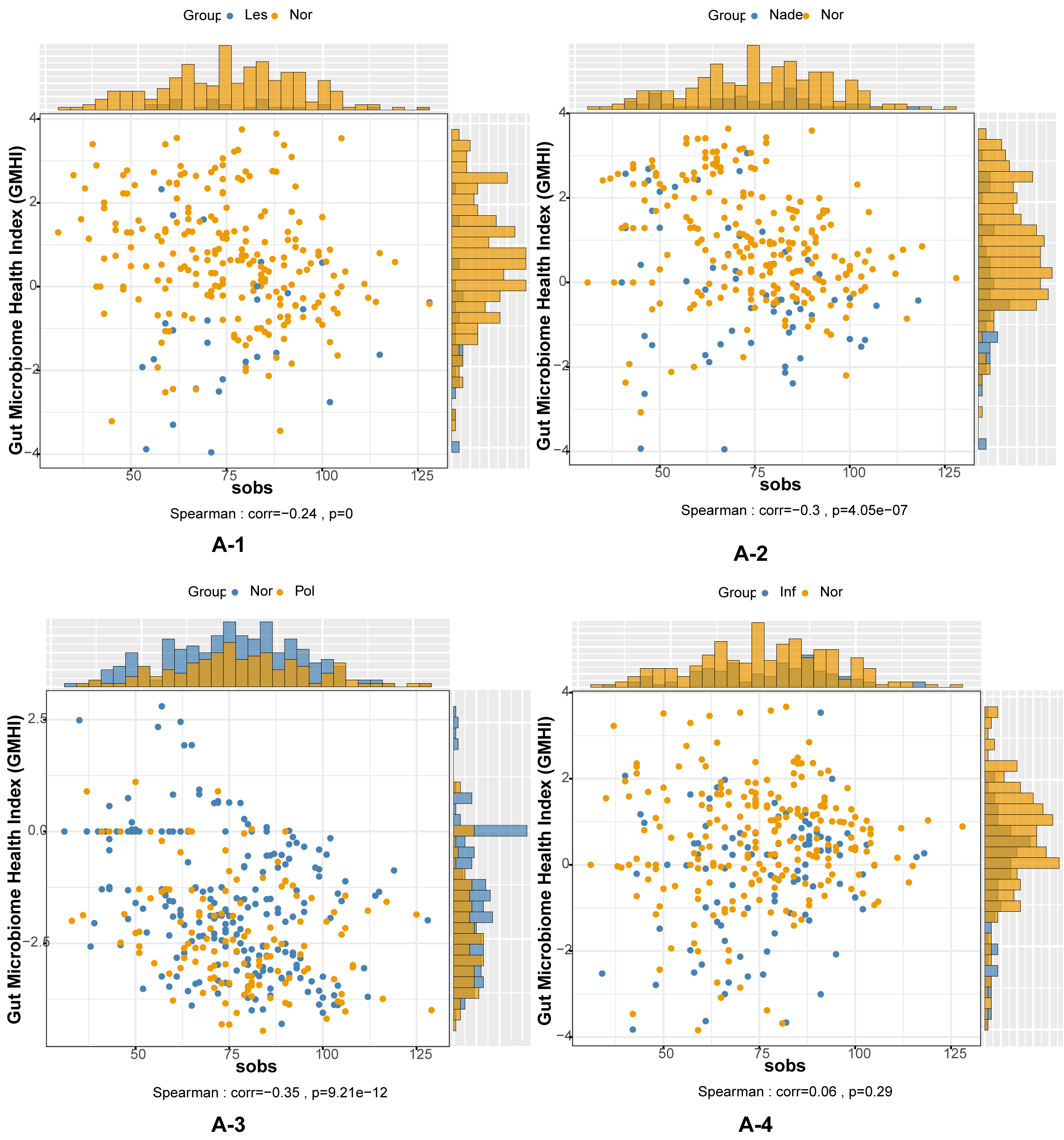


Figure 4 The correlation of GMHI and Alpha index between the four abnormal groups and control group


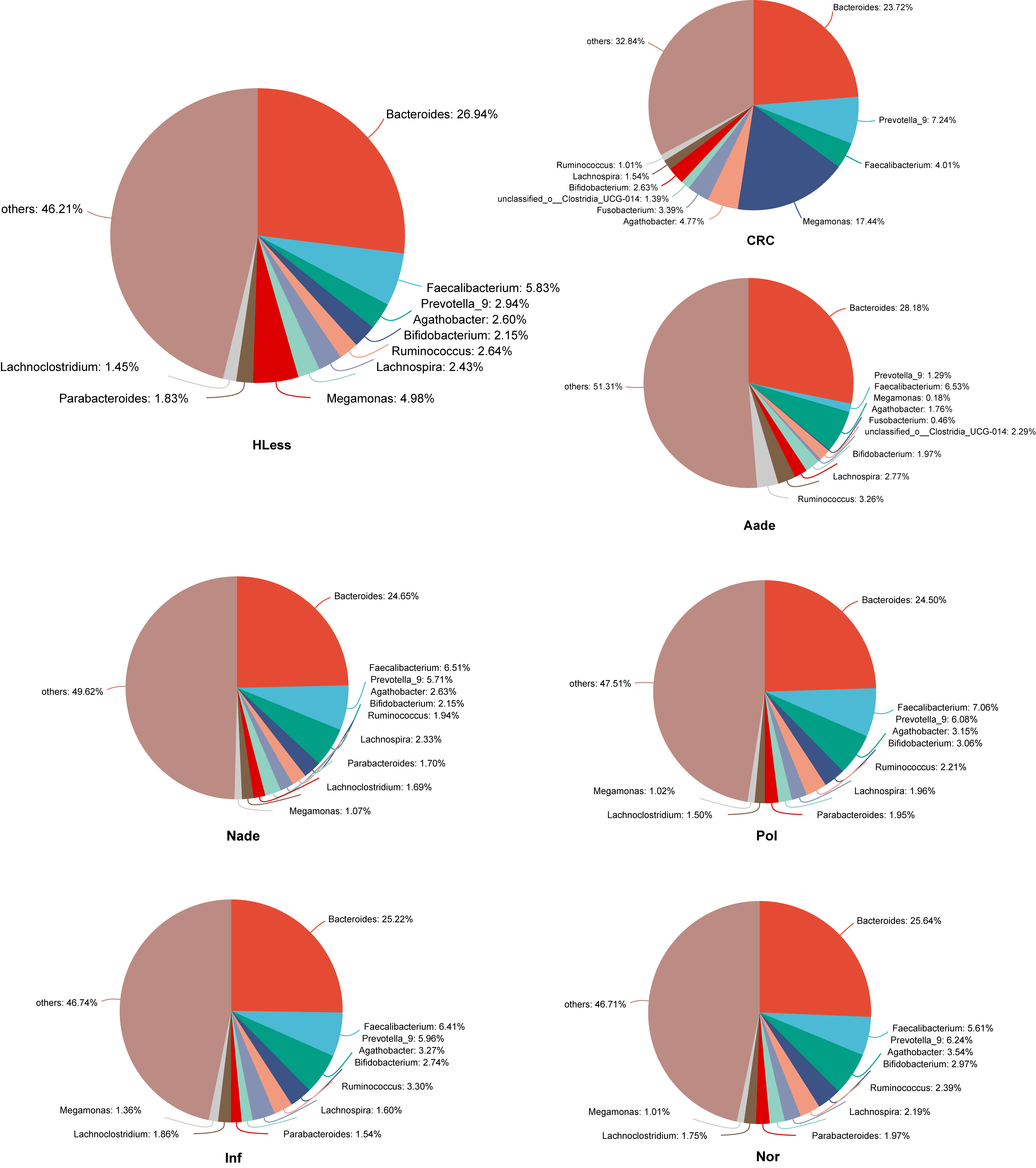


Figure 5 Community analysis pieplot on Genus level


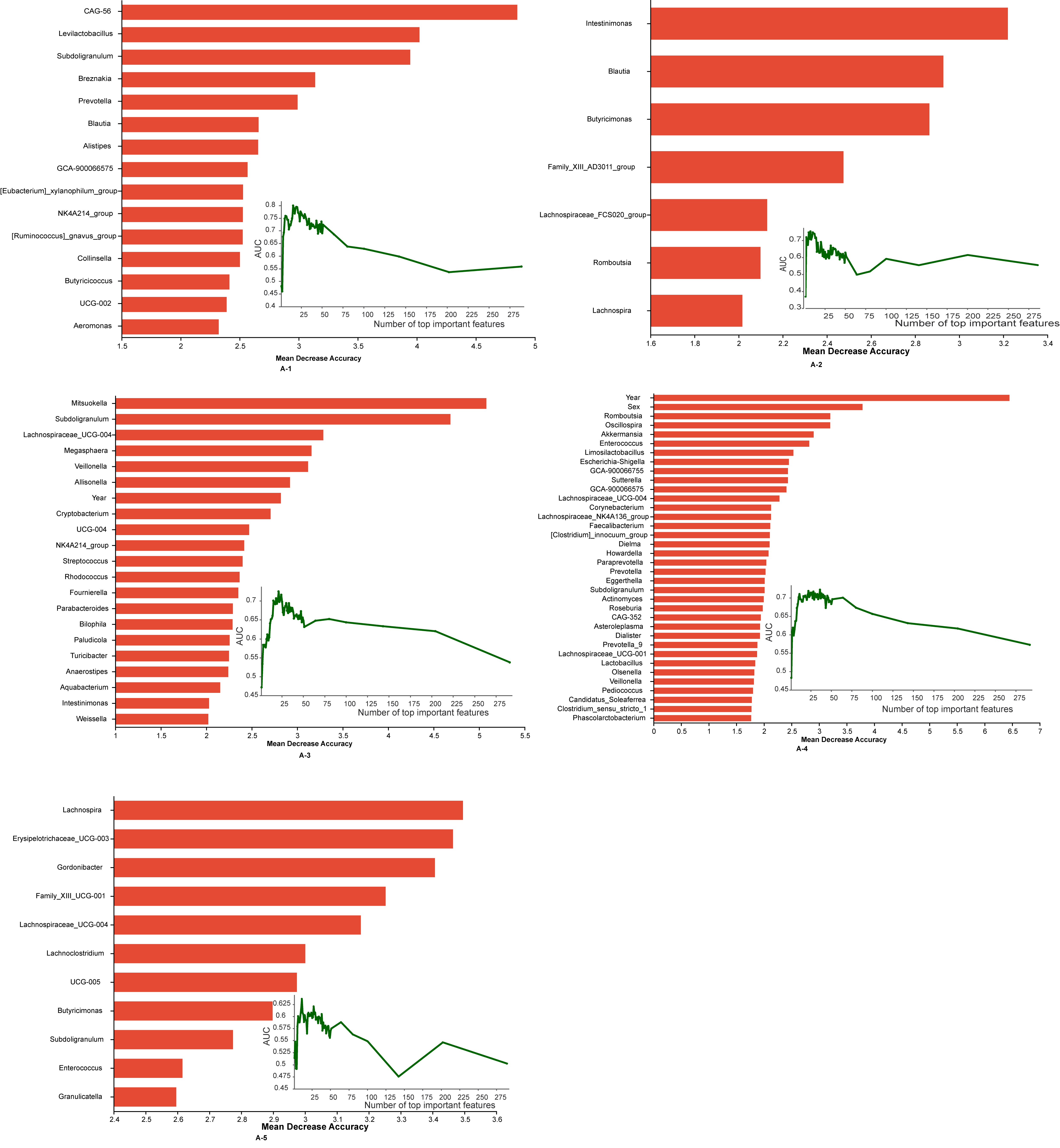


Figure 6 Bar plot illustrating the importance of variables at the genus level in the evaluation of Random Forest model, utilizing the most significant features. A1-A5 HLes & Nor, Aade & Nor, Nade & Nor, Pol & Nor, Inf & Nor
